# Homophilic protein interactions facilitate bacterial aggregation and IgG-dependent complex formation by the *Streptococcus canis* M protein SCM

**DOI:** 10.1080/21505594.2019.1589362

**Published:** 2019-03-24

**Authors:** Andreas Nerlich, Antje-Maria Lapschies, Thomas P. Kohler, Ingrid Cornax, Inga Eichhorn, Oliver Goldmann, Petra Krienke, Simone Bergmann, Victor Nizet, Sven Hammerschmidt, Manfred Rohde, Marcus Fulde

**Affiliations:** a Department of Internal Medicine/Infectious Diseases and Pulmonary Medicine, Charité Universitätsmedizin Berlin, Berlin, Germany; b Department of Medical Microbiology, Helmholtz Centre for Infection Research, Braunschweig, Germany; c Institute of Microbiology and Epizootics, Centre of Infection Medicine, Freie Universität Berlin, Berlin, Germany; d Department of Molecular Genetics and Infection Biology, Interfaculty Institute for Genetics and Functional Genomics, Center for Functional Genomics of Microbes, University of Greifswald, Greifswald, Germany; e Department of Pediatrics and Skaggs School of Pharmacy and Pharmaceutical Sciences, UC San Diego, La Jolla, CA, USA; f Infection Immunology Group, Helmholtz Centre for Infection Research, Braunschweig, Germany; g Department of Infection Biology, Institute of Microbiology, Technische Universität Braunschweig, Braunschweig, Germany; h Central Facility for Microscopy, Helmholtz Centre for Infection Research, Braunschweig, Germany

**Keywords:** *Streptococcus canis*, M protein, bacterial aggregation, protein complex formation

## Abstract

*Streptococcus canis* is a zoonotic agent that causes serious invasive diseases in domestic animals and humans, but knowledge about its pathogenic potential and underlying virulence mechanisms is limited. Here, we report on the ability of certain *S. canis* isolates to form large bacterial aggregates when grown in liquid broth. Bacterial aggregation was attributed to the presence and the self-binding activity of SCM, the M protein of *S. canis*, as evaluated by bacterial sedimentation assays, immunofluorescence- and electron microscopic approaches. Using a variety of truncated recombinant SCM fragments, we demonstrated that homophilic SCM interactions occur via the N-terminal, but not the C-terminal part, of the mature M protein. Interestingly, when incubated in human plasma, SCM forms soluble protein complexes comprising its known ligands, immunoglobulin G (IgG) and plasminogen (Plg). Co-incubation studies with purified host proteins revealed that SCM-mediated complex formation is based on the interaction of SCM with itself and with IgG, but not with Plg or fibrinogen (Fbg), well-established constituents of M protein-mediated protein complexes in human-associated streptococci. Notably, these soluble, SCM-mediated plasma complexes harbored complement factor C1q, which can induce complement breakdown in the periphery and therefore represent another immune evasion mechanism of SCM.

## Introduction

*Streptococcus canis* is a frequent colonizer of mucosal surfaces and the skin of dogs and cats, and occasionally identified in various other host species such as cows, rats, minks, mice, rabbits, and foxes [1–6]. As an opportunistic pathogen, *S. canis* infections generally lead to local and self-limiting alterations of skin and mucosa, but in some cases can proceed to severe and life-threatening diseases, such as streptococcal toxic shock-like syndrome (STSLS), necrotizing fasciitis (NF), meningitis and septicemia [7–10]. *S. canis* can be transmitted among different host species suggesting a certain zoonotic potential [11–16].

Streptococcal M proteins are important virulence factors that confer anti-phagocytic properties, but may also contribute to the development of post-streptococcal (autoimmune) sequelae [17,18]. *S. canis* was long considered to be “M protein negative”, since very few epidemiological studies reported about the identification of *emm*-typeable *S. canis* isolates [19,20]. However, we recently identified an open reading frame in a zoonotic strain of *S. canis*, which although not typeable according to the *emm*-typing scheme released by the Centers of Disease Control and Prevention (CDC), encoded a protein that shared important characteristics of streptococcal M proteins. We named this protein SCM for *S. canis* M protein [21]. SCM is a surface-attached, fibrillar protein which is dimerized under physiological conditions. Its anti-phagocytic activity is based on its particular capability to interact with itself (i) directly in a homophilic manner and (ii) indirectly using the host-derived zymogen plasminogen as a bridging molecule [22]. Furthermore, we recently described how SCM binds immunoglobulin G (IgG) in a non-opsonic manner (via the constant IgG-Fc domain) and found that this interaction prevents the deposition of C1q, a primary component of the classical complement activation pathway, on the bacterial surface [23].

The present manuscript describes the molecular mechanisms that underlie the self-binding capability of SCM and its consequences for the pathogenesis of *S. canis*. In particular, we demonstrate that SCM alone, when expressed on the bacterial surface, is sufficient for the generation of large bacterial aggregates. Bacterial aggregation is a common immune evasion mechanism against phagocytic killing and is usually mediated by bacterial surface proteins [24–27]. In other streptococci, bacterial self-aggregation has been attributed to M- and M-like proteins [28], but whether SCM, the M protein of *S. canis*, also induces bacterial clumping has not been previously investigated.

Lastly, we show that the soluble form of SCM leads to the formation of multimeric protein complexes upon incubation in human plasma. Homophilic SCM-SCM-interactions and concurrent IgG-binding activity are required for protein complex formation. Interestingly, and in contrast to surface-attached SCM [23], the interaction between SCM and IgG in complex enables C1q sequestration and may represent a virulence mechanism to evade complement-mediated opsonization.

## Results

### SCM mediates bacterial self-aggregation

**Figure 1.**
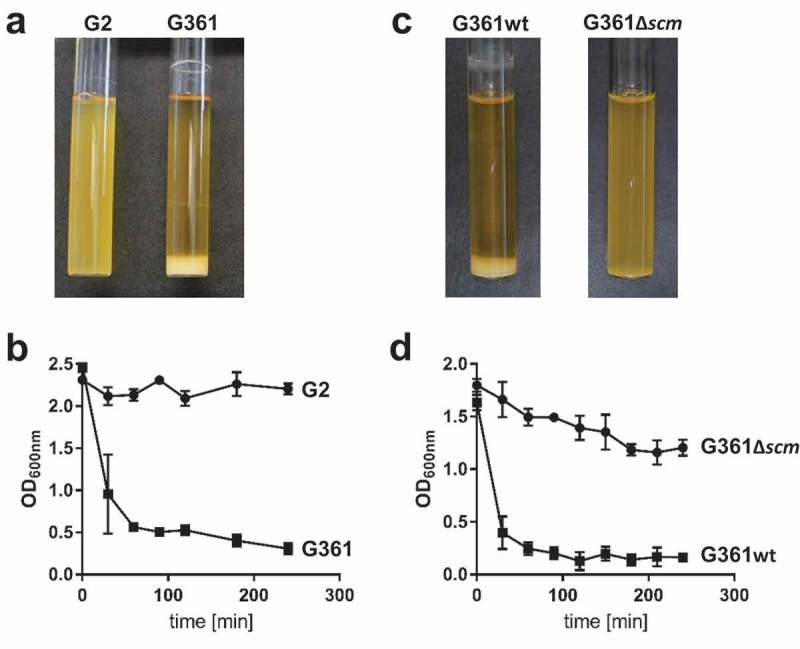
Analysis of streptococcal aggregation. (a) *S. canis* strain G2 (SCM^−^) and G361 (SCM^+^) were grown overnight at 37°C in TSB. The photograph shows bacterial sedimentation. (b) Quantification of the sedimentation rate of the bacterial cultures shown in (a) by measuring the optical density at 600 nm at the indicated time points. The results present mean and standard deviation of a representative experiment done in triplicates. The experiments were repeated three times. (c) *S. canis* strain G361 wildtype (G361) and the isogenic *scm*-targeted insertional mutant (G361∆*scm*) were grown overnight at 37°C in TSB. The photograph shows bacterial sedimentation. (d) Quantification of the sedimentation rate of the bacterial cultures shown in (c) by measuring the optical density at 600 nm at the indicated time points. The results present mean and standard deviation of a representative experiment done in triplicates. The experiments were repeated three times.

SCM-positive (SCM^+^) *S. canis* strain G361, its isogenic SCM-targeted insertional mutant (G361Δ*scm*), and naturally occurring SCM-negative (SCM^−^) *S. canis* isolate G2 were incubated overnight in liquid broth. As depicted in Figure 1(a,c), two different phenotypes were observed: whereas SCM-negative strains G2 and G361Δ*scm* grew as homogenous suspensions, broth cultures inoculated with SCM^+^ isolate G361 showed bacterial sedimentation at the bottom of the test tube and an almost clear supernatant. Re-suspension of the bacterial sediment of strain G361 was followed by an immediate, time-dependent re-sedimentation (Figure 1(b,d), squares). In contrast, neither shaking the homogenous cultures of the SCM^−^ strains G2 and G361Δ*scm* led to bacterial sedimentation nor to the clearance of the culture supernatants (Figure 1(b,d); circles). To test, whether SCM was not only necessary but also sufficient to promote sedimentation in liquid broth, we generated an SCM-expressing *Streptococcus gordonii* (SGO-SCM) strain by heterologous gene expression. As predicted, sedimentation experiments depicted in Figure S1 demonstrated an aggregative phenotype of SCM^+^ *S. gordonii*, whereas the SCM^−^ parental strain (SGO) grew homogenously in liquid broth.

**Figure 2.**
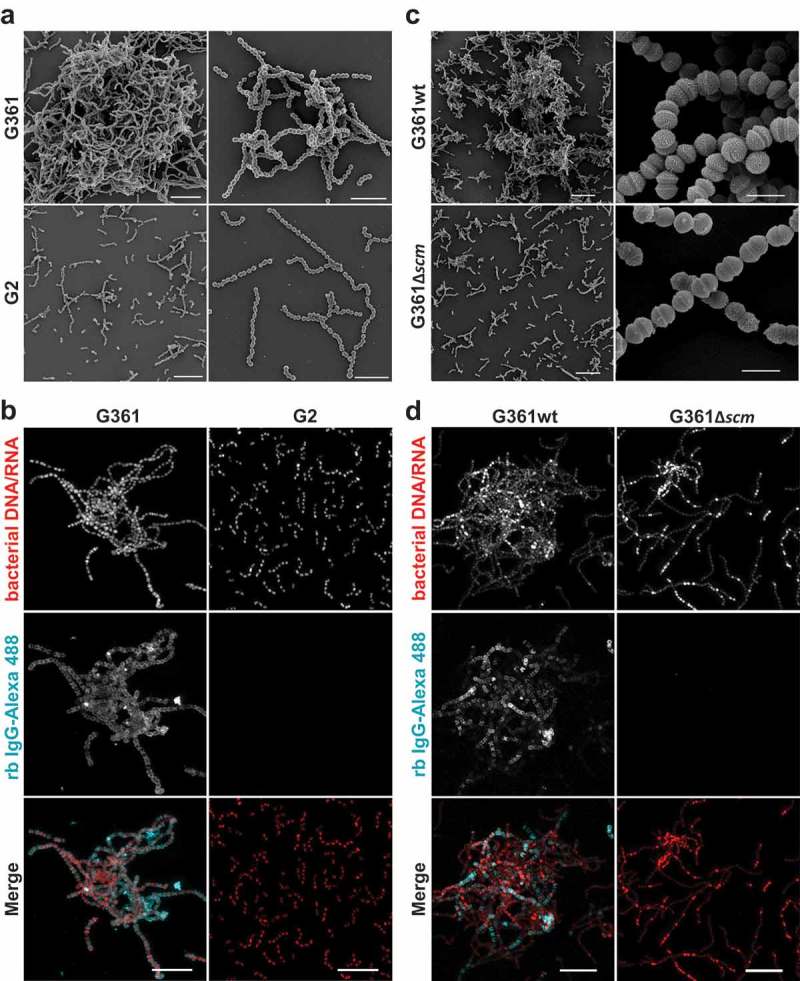
Microscopic analysis of streptococcal aggregation. (a) Scanning electron microscopic visualization of *S. canis* strain G2 and G361 from overnight cultures demonstrating bacterial aggregation of the SCM^+^ strain G361. Scale bars represent 10 µm left column and 5 µm right column. (b) Confocal microscopic analysis of bacterial aggregation and IgG binding. *S. canis* strain G361 and G2 were grown overnight at 37°C in TSB. Bacteria were allowed to adhere to poly-L-lysin-coated ibidi-slides, fixed and incubated with Alexa-488-conjugated rabbit (rb) IgG (cyan). Bacterial DNA/RNA was stained with ethidium homodimer-1 (red). Bacterial aggregation was visualized by confocal microscopy. Representative maximum intensity projections of deconvolved confocal z-stacks from 2 independent experiments are shown. Scale bar represents 10 µm. (c) Scanning electron microscopic visualization of *S. canis* strain G361wt and G361∆*scm* from overnight cultures demonstrating bacterial aggregation of the SCM^+^ strain G361wt. Scale bars represent 10 µm left column and 1 µm right column. (d) Confocal microscopic analysis of bacterial aggregation and IgG binding. *S. canis* strain G361wt and G361∆*scm* from overnight cultures generated and stained as described in (b). Alexa-488-conjugated rabbit (rb) IgG is shown in cyan and bacterial DNA/RNA stained with ethidium homodimer-1 is shown in red. Representative maximum intensity projections of deconvolved confocal z-stacks from 2 independent experiments are shown. Scale bar represents 10 µm.

To visualize bacterial aggregation, we conducted both, field emission scanning electron microscopy (FESEM) and confocal immunofluorescence microscopy (IF). Consistent with results obtained in the sedimentation assays, we found large bacterial aggregates of SCM^+^ strain G361 (Figure 2, Figure S2) that were entirely absent in bacterial cultures of strains lacking SCM (Figure 2). Notably, single bacteria in the large aggregates of strain G361 were decorated with IgG; no such binding was seen in the SCM^−^ strain G2 and the isogenic SCM-targeted mutant G361Δ*scm* (Figure 2). These results corroborate the role of SCM as the only IgG-binding receptor of *S. canis* [23].

### The N-terminal part of SCM mediates homophilic protein interactions

**Figure 3.**
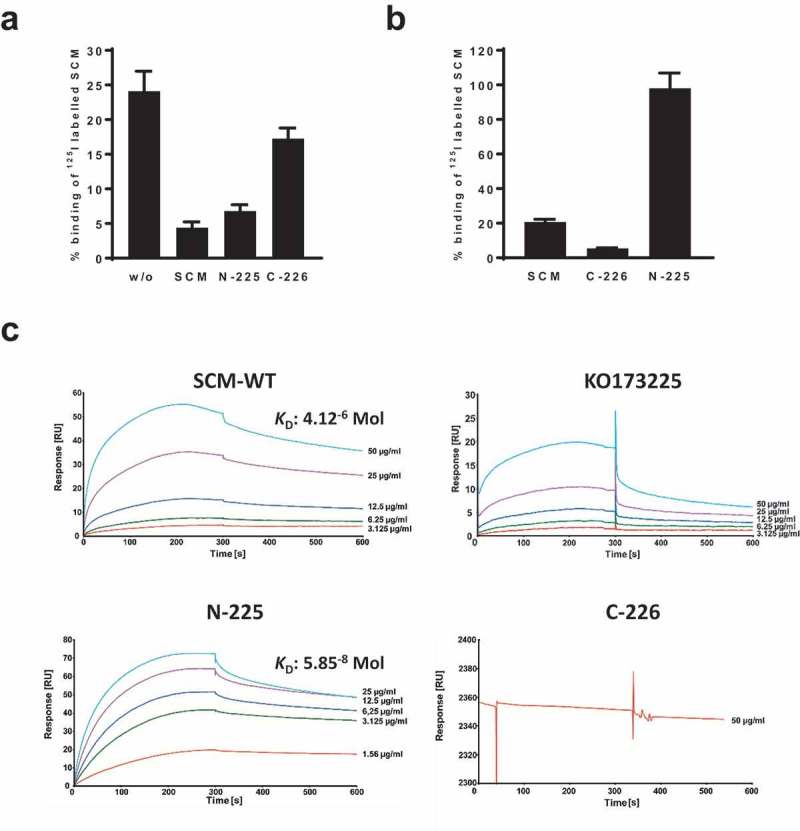
SCM interacts with itself via its N-terminus. (a) The SCM^+^ *S. canis* strain G361 was incubated with iodinated full-length SCM either without (w/o) or with pre-incubation with 1 µM non-iodinated SCM, N-225 or C-226, respectively. (b) *S. canis* G 361 was co-incubated with iodinated SCM and its derivatives C-226 and N-225, respectively. Results are depicted as mean and SD of percentage binding of totally used iodinated protein. (c) Interactions of soluble SCM-WT and its truncated fragments KO173225, N-225 and C-226, respectively, with immobilized SCM-WT were analyzed by surface plasmon resonance spectroscopy. Representative sensorgrams of three independent experiments show a dose-dependent binding of SCM-WT, KO173225 and N-225. No binding was observed for the C-terminal fragment C-226. The association and dissociation was observed, each of 300 s. Values of the control flow cells were subtracted from each sensorgram. The *K*_D_ value is indicated for the interaction between SCM and SCM. A representative *K*_D_ value was calculated for the interaction between N-225 and SCM.

Since SCM self-interactions are reported [22], we probed the role of this phenomenon in more detail. First, we conducted binding inhibition studies incubating strain SCM^+^ G361 with iodinated recombinant SCM and different truncated, non-labelled SCM fragments (Figure 3(a)). In the absence of unlabelled protein, the SCM binding capacity of strain G361 was determined to be 24.1% ± 2.9%. Supplementation with 5 µg non-labelled WT SCM considerably decreased binding to 4.4% ± 0.8%. A similar reduction was observed using fragment N-225 comprising the first 225 amino acids of the mature SCM protein (6.8% ± 0.9%). In contrast, using the C-terminal fragment C-226, a binding capacity of 17.3% ± 1.5% was detected which was similar to the mature SCM protein. To verify these data, we iodinated truncated SCM fragments N-225 and C-226, and incubated them with SCM^+^ strain G361 (Figure 3(b)). As predicted, the C-terminal fragment C-226 exhibited only marginal affinity to strain G361, whereas a strong interaction was detected using fragment N-225 (Figure 3(b)).

To quantify the self-interaction of SCM, we performed surface plasmon resonance analysis using the Biacore® T200 system. Recombinant SCM was immobilized on a CM5 sensor chip as a ligand and mature WT as well as different truncated SCM fragments were applied as analytes in a series of concentrations (1.5–50.0 µg/ml). The sensorgrams for SCM-WT, N-225, and KO173225 showed specific and dose-dependent binding to SCM, whereas no binding was detected for the C-terminal fragment C-226 (Figure 3(c)). The dissociation constant for the binding of SCM to SCM was calculated from three independent experiments as 4.12 × 10^−6^ Mol. Notably, a representative dissociation constant of 5.85 × 10^−8^ Mol was determined for the interaction between SCM and N-225. In summary, these data clearly show that SCM binds to itself via the N-terminal part of the mature protein.

### SCM leads to the formation of protein aggregates in human plasma

**Figure 4.**
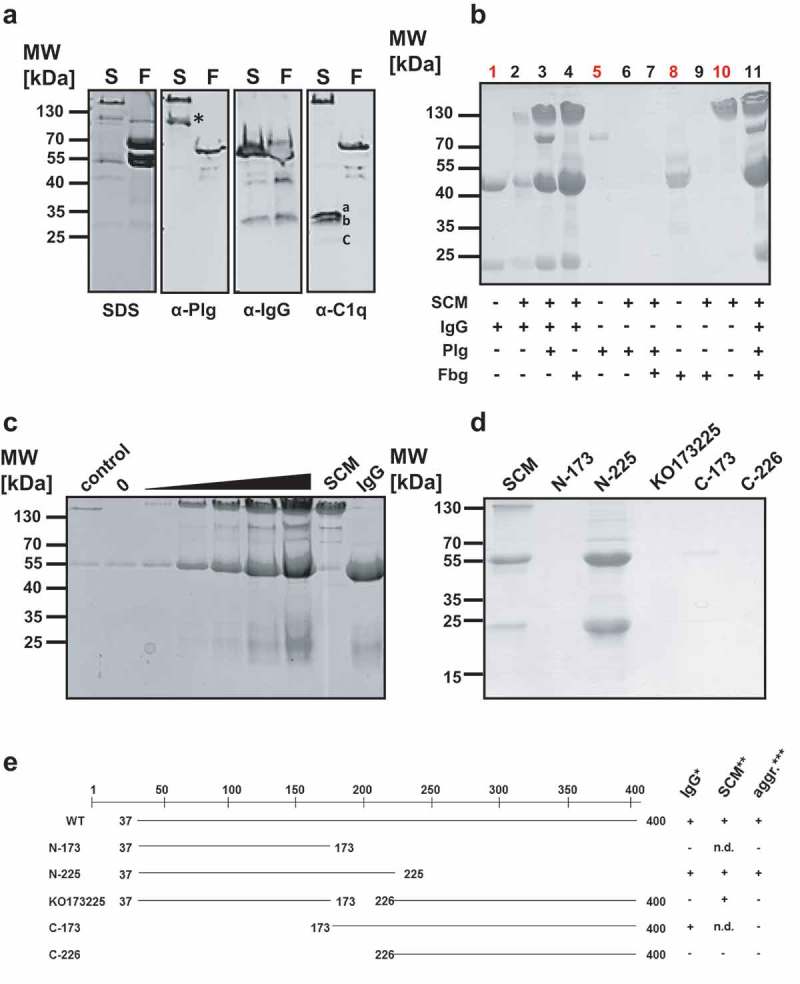
SCM-mediated complex formation is IgG-dependent. (a) Recombinant full-length SCM protein from strain G361 was added to human plasma (1:10 diluted) and incubated for 4 h at 37°C. After precipitation, protein aggregates were carefully washed with PBS, resuspended in Laemmli buffer and separated on a 10% SDS gel (SDS) or blotted onto a PVDF membrane and applied for western blot analysis using antibodies directed against plasminogen (α-Plg), IgG (α-IgG), and C1q (α-C1q). The three subunits of C1q (a, b, c) are indicated. (b) Recombinant WT-SCM was co-incubated with different host proteins alone or in combination and tested for complex formation (black numbers). Human IgG (1), plasminogen (5), fibrinogen (8) and recombinant SCM (10) served as protein quality controls (red numbers). (c) Increasing amounts of recombinant SCM protein (1 µg – 80 µg) were co-incubated with 20 µg human IgG. The resulting protein aggregates were analysed by SDS-PAGE. 20 µg of the truncated SCM fragment lacking the IgG binding region (KO173225) served as a control. 0, 20 µg IgG was incubated without SCM supplementation. (d) Recombinant full-length protein SCM and its derivatives N-173, N-225, KO173225, C-173 and C-226, respectively, were incubated with 1:10-diluted human plasma. Complex formation was monitored qualitatively by SDS-PAGE. (e) Schematic representation of the truncated fragments used in this study and their ability to bind IgG, SCM and to induce complex formation.

The incubation of SCM with human plasma led to formation of soluble protein aggregates, which were subsequently harvested and separated by SDS-PAGE. Several protein bands were detected with molecular masses ranging from approximately 25 kDa to 160 kDa (Figure 4(a); SDS). We next applied western blot analysis of SCM-mediated protein complexes from human plasma to investigate their composition in more detail. In a first step, we used antibodies against known SCM ligands, such as Plg and IgG [21–23]. We were also interested in knowing whether complement factor C1q is incorporated into the protein aggregates, since this might constitute another immune evasion pathway for *S. canis*. For comparison, we included the fibrinogen binding protein of G streptococci (FOG), an M protein of human-adapted *S. dysgalactiae* subsp. *equisimilis* (*S. equisimilis*), which forms protein aggregates upon co-incubation with human plasma [29]. As depicted in Figure 4(a), both SCM (S) and protein FOG (F) led to complex formation upon incubation in human plasma as demonstrated by SDS-PAGE (SDS). In western blot analysis, a specific band of approximately 92 kDa was observed for SCM- (S, asterisk), but not for FOG-mediated protein complexes (F), when antibodies directed against human Plg (α-Plg) were used. Because of the intrinsic IgG binding capability of SCM [23] and FOG [30], protein bands with molecular masses of approximately 160 kDa (S) and 66 kDa (F) appeared, resulting from the non-opsonic interaction between both M proteins and the secondary antibody. To verify the presence of IgG molecules in the protein complexes, we used a secondary antibody against human IgG (α-IgG) that was raised in goats and therefore not recognized by the IgG-binding regions of SCM and FOG [23,30]. This antibody detected the presence of IgG heavy and light chains in both, SCM- and FOG-mediated protein complexes. However, whether IgG was necessary for complex formation in FOG-derived protein aggregates (as shown for SCM), could not be finally clarified. Interestingly, C1q, an IgG ligand and an early component of the classical complement pathway was detected only in SCM-, but not in FOG-mediated protein complexes (α-C1q).

To investigate the nature of the SCM-mediated complex formation in more detail, we co-incubated SCM with its ligands IgG and Plg as well as fibrinogen (Fbg), since Fbg promotes complex formation with M proteins from *S. pyogenes* (Group A S*treptococcus*, GAS) and *S. equisimilis* [29,31]. As depicted in Figure 4(b), neither incubation of SCM with plasminogen (lane 6) nor incubation of SCM with Fbg (lane 9) or Plg and Fbg (lane 7) resulted in protein precipitation. However, co-incubation of SCM with IgG (lane 2) and, in particular, with the constant crystallisable part (F_c_) of the IgG molecule (IgG-F_c_), was not only sufficient but also necessary to facilitate protein aggregate formation (Figure S3). Notably, supplementation of additional plasma proteins, such as Plg and/or Fbg did not lead to inhibitory effects on complex formation (Figure 4(b), lanes 3, 4, 11). Rather, Plg (a further ligand of SCM), co-precipitated with SCM and IgG (Figure 4(b), lanes 3 and 11).

We next incubated a constant amount of IgG (20 µg) with different amounts of SCM (1, 10, 20, 40, and 80 µg) and analysed complex formation qualitatively by SDS-PAGE. As depicted in Figure 4(c), increasing concentrations of SCM concomitantly led to an increase in recruited IgG, suggesting that complex formation follows a strict stoichiometrical ratio. Notably, similar results were observed when different amounts of SCM were incubated with human plasma (Figure S4). Finally, protein aggregation assays were performed using different truncated SCM fragments with either IgG- (C-173), SCM- (KO173225), IgG- and SCM- (N-225) or neither IgG- nor SCM- (C-226) binding capabilities (Figure 4(d)). As expected, complex formation was only observed for the mature WT SCM and the N-terminal fragment N-225 that resembles both, SCM and IgG binding capability. Truncated fragments harbouring only SCM- or only IgG-binding activity did not form SCM-IgG protein complexes. An overview summarizing these results is given in Figure 4(e).

## Discussion

M- and M-like proteins constitute a large family of streptococcal surface proteins and numerous publications that summarize the current literature about their distribution and their function are available. One of the most important features of M- and M-like proteins is their anti-phagocytic property, which by definition has been confirmed in M proteins, but only assumed for M-like proteins [32]. The mechanisms by which M proteins facilitate anti-phagocytosis are versatile and include interactions with the host immune system (e.g. binding to plasma and extracellular matrix proteins, reviewed in [33,34]) but also the ability to interact with itself (homophilic protein interactions). Knowledge about the molecular mechanisms of the latter are scant and prompted us to conduct the present study.

We recently identified SCM, the M protein in *S. canis*, and confirmed its anti-phagocytic properties mediated by plasma protein binding [21–23]. SCM forms homophilic protein interactions and the supplementation of purified SCM protein to SCM-deficient *S. canis* significantly increased bacterial survival in co-incubation experiments with purified polymorphonuclear neutrophils (PMNs). In line with this, Frick and colleagues reported about homophilic protein interactions of the surface-associated proteins Protein H and M1 of GAS that led to bacterial aggregation and facilitated anti-phagocytosis by particle enlargement [28]. Similarly, we observed the formation of large bacterial aggregates in *S. canis* liquid cultures and demonstrated that SCM alone is not only sufficient but also necessary for this particular phenotype (Figures 1 and 2, Figures S1 and S2). However, whether or not bacterial aggregation is also important for *S. canis* to colonize the mucosal surfaces of the host remains unknown. Frick *et al*. found that GAS adhere significantly better to human epithelial cells when clustered in bacterial aggregates [28]. Caparon and colleagues proposed that bacterial aggregation on epithelial surfaces precedes microcolony formation [35], a well-established mechanisms of bacteria to establish themselves in hostile conditions.

Several streptococcal M- and M-like proteins are liberated from the bacterial surface by proteolytic cleavage and lead to complex formation when incubated in host plasma [36,37]. We have previously demonstrated that SCM is also released from the surface of *S. canis* [22], and the results of the present study show further that it forms similar soluble protein aggregates in the presence of host plasma. However, in contrast to M1 and FOG, co-incubation of SCM with fibrinogen did not lead to the formation of soluble protein aggregates. This finding is consistent with the lack of fibrinogen binding capability of SCM-positive strain G361 and in *S. gordonii* heterologously expressing SCM (Figure S5). However, co-incubation of SCM with its ligand IgG led to the formation of soluble protein aggregates (Figure 4(b)). Notably, a strong IgG-binding capability alone is not sufficient to induce complex formation, because truncated C-terminal SCM fragments exhibiting even stronger affinities for IgG [23], failed to induce protein aggregation when co-incubated with IgG. In contrast, N-terminal SCM fragments facilitate complex formation as long as they consist of the entire IgG-binding region (Figure 4(d)). These observations suggest that the generation of SCM-IgG complexes requires not only a functional IgG-binding site (and, thus, IgG-binding activity), but also the ability for SCM to interact with itself. Furthermore, our results clearly indicated that the region that mediates SCM self-binding activity is located in the N-terminal part of the mature protein. This conclusion is supported by the observation that (i), only the N-terminal fragment reduced the binding of iodinated SCM to the surface of *S. canis* in competition experiments, whereas the C-terminal part of SCM did not (Figure 3(a)), and (ii) radiolabelled SCM fragments showed only a strong back-binding activity to the surface of *S. canis* for the N-terminal SCM fragment, but not for C-226 (Figure 3(b)). Notably, the back-binding activity of N-225 to the surface of G361 was more pronounced than of the mature SCM protein, which was additionally confirmed in SPR analysis (Figure 3(c)). The calculated dissociation constant of 5.85 × 10^−8^ Mol for the interaction between SCM and N-225 exceeds the *K*_D_ of the SCM-SCM interaction (4.12 × 10^−6^ Mol) by approximately two logs. The reason is unknown, but intra-molecular structural differences between N- and C-terminal parts of the entire M protein are likely to be involved [38]. Interestingly, Protein H of GAS interacts with itself via a 18mer peptide region (designated AHP, for aggregative protein H peptide) located in the N-terminus [28]. AHP and similar peptide sequences were also identified in a series of other M- and M-like proteins from GAS, emphasizing the role of homophilic interactions in such alpha helical proteins. However, *in silico* analysis of AHP and the N-terminal part of SCM did not reveal any significant sequence homologies, neither at genomic nor protein levels (data not shown), suggesting the existence of analogous binding sites and again stressing the possibility of a convergent evolution of M- and M-like proteins [39].

An important, unsolved question is the biological role of protein complex formation. For the SCM analogue Protein H, when immobilised on the bacterial surface, interaction with IgG inhibits the deposition of C1q and C3, which led to a significantly reduced complement-mediated opsonisation and immune-cell-mediated killing of the bacteria. In contrast, the presence of C1q in M-protein-mediated IgG complexes led to complement breakdown distant from the bacterial surface, thus representing another virulence trait of IgG-Fc receptor positive streptococci [40]. We similarly observed an inhibition of C1q deposition on SCM^+^ *S. canis* isolates [23] and a sequestration of C1q in SCM-plasma complexes (Figure 4) implicating that SCM is likewise involved in the resistance against complement-mediated killing by phagocytes. In addition to interference with complement, Herwald and colleagues reported that Protein M1 from GAS forms complexes with fibrinogen when added to human serum [41]. These protein complexes, in turn, activate PMNs to release the heparin binding protein that might ultimately lead to vascular leakage, shock and multi-organ failure. Notably, these clinical pictures are not pathognomonic for systemic infections of human-restricted streptococci, but are also found in cats suffering from invasive *S. canis* infections [8,9,42–44].

In summary, we investigated the molecular mechanisms behind the aggregative phenotype of SCM^+^ *S. canis* strains. We found that anti-phagocytic M protein SCM mediates homophilic protein interactions that, when immobilized on the bacterial surface, led to the formation of large bacterial aggregates. In the presence of IgG, SCM protein, which is liberated from the bacterial surface, triggers the formation of soluble protein complexes composed of various host-plasma proteins, including components of the complement system. However, whether bacterial aggregation and SCM-mediated complex formation impacts the outcome of mucosal colonization or systemic dissemination in the pathogenesis of *S. canis* infections remains unknown and awaits further investigations.

## Material and methods

### Bacterial strains and growth conditions

The SCM-positive *Streptococcus canis* strain G361 (kindly provided by Dr. Mark van der Linden, National Reference Centre for Streptococci, RWTH Aachen, Aachen, Germany) was introduced in several studies [6,21–23,45] and its genome was released only recently [46]. G361 was isolated from a vaginal swab of a 40-year-old woman. *S.* *canis* strain G361 is not typeable by the CDC *emm*-typing scheme. The isolation site of strain G2 is unknown. *S. gordonii* heterologously expressing SCM and its SCM-negative parental strain was described earlier [21]. Bacteria were routinely grown in tryptic soy broth (TSB) or Todd-Hewitt broth (THB) at 37°C without shaking. In preparation for electroporation, G361 was grown overnight in THB supplemented with 0.6% glycine and subcultured into 50 ml of THB the following day. The resulting culture was harvested at 1.5 hours of culture by centrifugation (10 min at 3250 x *g*), washed once in 50 ml 0.625 M sterile sucrose solution, and resuspended in 250 μl of 0.625 sucrose solution. Fifty μl aliquots were kept frozen at −80ºC until needed. *Escherichia coli* strains were cultivated in Luria-Bertani medium. Where indicated, antibiotics were supplemented as follows. *E. coli*: ampicillin (100 μg/ml), kanamycin (25 μg/ml), erythromycin (Erm) (300 μg/ml); *S. gordonii*: Erm (1 μg/ml); G361Δ*scm* Erm (2 μg/ml).

### Antibodies and reagents

Human IgG, papain-treated fragments of human IgG, human plasminogen and fibrinogen were purchased from Sigma. Polyclonal antibodies against human IgG and plasminogen as well as HRP-conjugated anti-human antibodies were purchased from Dako. Antibodies directed against human C1q were from Calbiochem. Ethidium homodimer-1 and the secondary rabbit anti-mouse IgG Alexa Fluor 488 conjugated antibody (Cat#: A-11059; RRID: AB_2534106) were obtained from Life Technologies. 16% formaldehyde without methanol and CitiFluor™ CFM3 mounting medium were obtained from Electron Microscopy Science.

### Construction of the G361 scm targeted insertional mutant

Targeted insertional mutagenesis of *scm* was performed as previously described [47] using the pHY304 vector [48]. Briefly, an intragenic fragment from the *scm* gene was amplified by PCR using the following primer pair: TCTCAAGCTTTGACGGAGCAAG; TCTTCTAGATCAGCTGTCAAGCG. PCR products were recovered by T-A cloning in the vector pCR2.1-TOPO (Invitrogen) and then were cloned by *Hin*dIII/*Xba*I digestion into the temperature-sensitive vector pHY304. Three μg of the resultant knockout plasmid was introduced into G361 by electroporation, and Erm-resistant transformants were identified at the permissive temperature for plasmid replication (30°C). Single-crossover Campbell-type chromosomal insertion was selected by shifting to the nonpermissive temperature (37°C) while maintaining Erm selection. SCM phenotype was determined on Todd-Hewitt agar (THA) plus 2 μg Erm at 37°C.

### Cloning and expression of truncated SCM fragments

Cloning and overexpression of recombinant SCM as well as its truncated fragments was already introduced earlier [21,23]. Briefly, based on the mature *scm* fragment that was cloned into the Qiagen´s pQE30 expression vector, an inverse PCR technique was developed that, based on the positioning of the oligonucleotides, the generation of N-terminal, C-terminal and internal deletion fragments. An overview about the truncated SCM fragments that were used in the present paper are given in Figure 4(e). Overexpression and purification of histidine-tagged recombinant proteins were done essentially as described by the manufacturer (Qiagen Expressionist^TM^ System).

### Protein aggregation assays, SDS-PAGE and western blot analysis

Various amounts of recombinant FOG [30], SCM [21] or its truncated fragments N-173, N-225, C173, C-226 and KO173225 [23], respectively, were incubated in a 1:10 dilution (in PBS) of human plasma for 4 hours at 37°C in a water bath. After centrifugation at 13,000 rpm, protein aggregates were washed two times in 100 µl 0.1 M NaCl containing 0.5% (v/v) Triton X-100 and finally resuspended in 20 µl Laemmli buffer. Co-incubation of SCM with purified proteins (IgG, Plg and Fbg, respectively) was carried out in PBS containing 2% (v/v) Triton X-100. All protein suspensions were incubated for 1 h at room temperature. Centrifugation steps and washing procedures were as described above. In any case, protein complexes were separated by a 10% reducing SDS gel. If indicated, the resuspended protein complexes were subjected to immunoblotting using the BioRad semidry system essentially as described earlier [21]. Primary antibodies against Plg, IgG and C1q, respectively, were used in the following dilutions: α-Plg, 1:5000; α-IgG, 1:2000; α-C1q, 1:1500. HRP-conjugated secondary antibodies were used in a 1:3000 dilution. Peroxidase activity was detected by chemoluminescence using 100 mM Tris HCl, 1.25 mM 3-aminopthalhydrazide, 225 µM p-coumaric acid, and 0.01% H_2_O_2_ at pH 8.8 in water and exposed to chemoluminescence films (Hyperfilm; Amersham).

### Field emission scanning electron microscopy (FESEM)

Bacteria were fixed with 5% formaldehyde and 2% glutaraldehyde in growth media, left overnight at 7°C, and then washed with TE buffer (10 mM TRIS, 2mM EDTA, pH 6.9). Samples were dehydrated in a graded series of acetone (10, 30, 50, 70, 90, 100%) on ice for 10 min for each step. Samples in the 100% acetone step were allowed to reach room temperature before another change in 100% acetone. Samples were then subjected to critical-point drying with liquid CO_2_ (CPD 030, Bal-Tec,). Dried samples were coated with a gold/palladium (80/20) film by sputter coating (SCD 500, Bal-Tec) before examination in a field emission scanning electron microscope Zeiss Merlin using the Everhart Thornley HESE2-detector and the inlens SE-detector in a 25:75 ratio at an acceleration voltage of 5 kV. Images were recorded with Zeiss SEMSmart V 5.05 and contrast and brightness were adjusted with Adobe Photoshop CS5.

### Immunofluorescence staining and confocal microscopy

For confocal microscopy, 100 µl of bacterial overnight culture was added into poly-L-lysine-coated µ-slide 8-well (ibidi) filled with 100 µl PBS for 30 min. Subsequently, specimens were fixed with 3% formaldehyde for 15 min. Following washing with PBS samples were incubated with rabbit anti-mouse Alexa Fluor 488-conjugated IgGs in PBS/1% BSA/Tween 20 overnight at 4°C. Bacterial RNA/DNA was stained with ethidium homodimer-1 and samples were embedded in CitiFluor™ CFM3 mounting medium. Mounted samples were examined by confocal microscopy as described previously [49]. In brief, image stacks with a z-step size of 0.2 μm per plane were acquired using a 63 × /1.4 NA Plan-Apochromat objective. The pinhole was set to 1 airy unit and images were acquired in sequential imaging mode to avoid bleed-through of fluorescence emission. Images were deconvolved using Huygens® Essential 15.10 (Scientific Volume Imaging) and 3D-stacks are displayed as maximum intensity projections (MIPs) and adjusted identically for brightness and contrast in ImageJ/Fiji [50]. 3D-rendering was done with ParaView v5.4 (Kitware Inc.).

### Surface plasmon resonance spectrometry

The interaction of SCM-WT with itself as well as with truncated fragments was analysed by surface plasmon resonance spectroscopy using a Biacore T200 optical biosensor (GE Healthcare). Therefore, recombinant SCM-WT was immobilized on a carboxymethyl dextran sensor chip (CM5) essentially as described previously [23]. SCM-WT, KO173225 and the C- and N-terminal fragments N-225 and C-226, respectively, were used as analytes in a concentration range of 1.56–50 µg/ml. Binding analysis was performed in PBS containing 0.05% Tween 20 at 25°C and a flow rate of 10 µl/min. Data were analysed using Biacore T200 evaluation software (version 2.0.1.1).

### Radioactive labelling, binding and inhibition studies

Iodination of fibrinogen, SCM and its truncated fragments SCM-N225 and SCM-C226 was performed with the chloramine T method as described earlier [51]. Binding and subsequent inhibition were carried out as described elsewhere [21,23].

## Supplementary Material

Supplemental Material

## Abbreviations

AHP: aggregative protein H peptide
BSA: bovine serum albumin
CDC: Center of Disease Control and Prevention
DNA/RNA: deoxyribonucleic acid/ ribonucleic acid
Fbg: fibrinogen
Fig: Figure
FOG: Fibrinogen-binding protein of G streptococci
FgBP: Fibrinogen-binding protein
GAS: Group A streptococcus
HRP: horseradish peroxidase
IgG: Immunoglobulin G
kDa: kilo Dalton
MIP: maximum intensity projection
NF: necrotizing fasciitis
OD: optical density
PBS: phosphate buffered saline
PCR: polymerase chain reaction
Plg: plasminogen
PMN: polymorphonuclear neutrophil
*S*: *Streptococcus*
SCM: *Streptococcus canis* M protein
SDS-PAGE: sodium dodecyl sulfate polyacrylamide gel electrophoresis
SEM: scanning electron microscopy
SGO: *Streptococcus gordonii*
SPR: surface plasmon resonance
STSLS: Streptococcal toxic shock-like syndrome
TSB: tryptic soy broth
WT: wild type
