# Supplementary material for: Homophilic protein interactions facilitate bacterial aggregation and IgG-dependent complex formation by the Streptococcus canis M protein SCM
Source: Virulence. 2019 Mar 24;10(1):194–206. doi: 10.1080/21505594.2019.1589362 (PMC6527014; doi:10.1080/21505594.2019.1589362)
Supplement: Supplemental Material [file kvir-10-01-1589362-s001.pdf]

Supplementary Figure 1

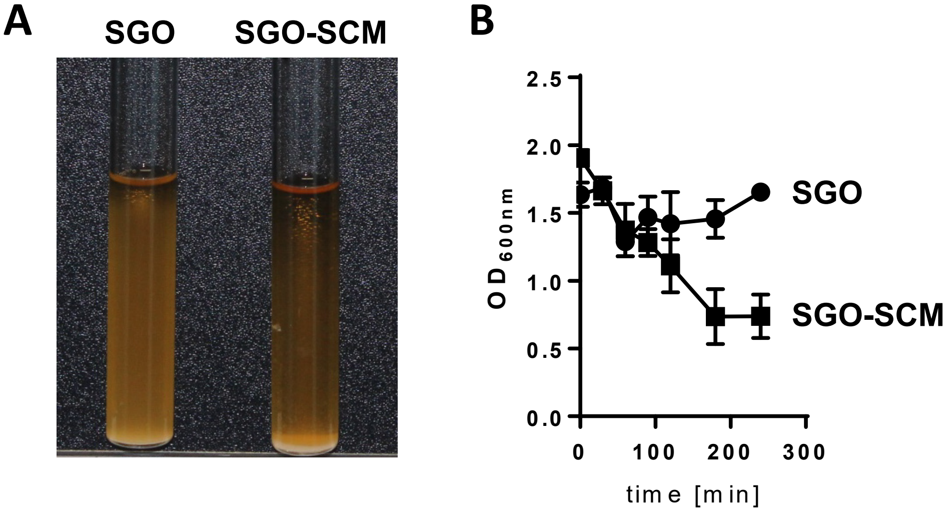

Supplementary Figure 1. Analysis of streptococcal aggregation. (**A**) *S. gordonii* wild type strain (SGO) and SGO expressing SCM (SGO-SCM) were grown overnight at 37°C in TSB. Bacterial sedimentation was visualized by a photograph. (**B**) Quantification of the sedimentation rate of the bacterial cultures shown in (A) by measuring the optical density at 600 nm at the indicated time points. Data represent mean ± SD from a representative experiment performed in triplicates. Experiments were repeated at least three times.

Supplementary Figure 2

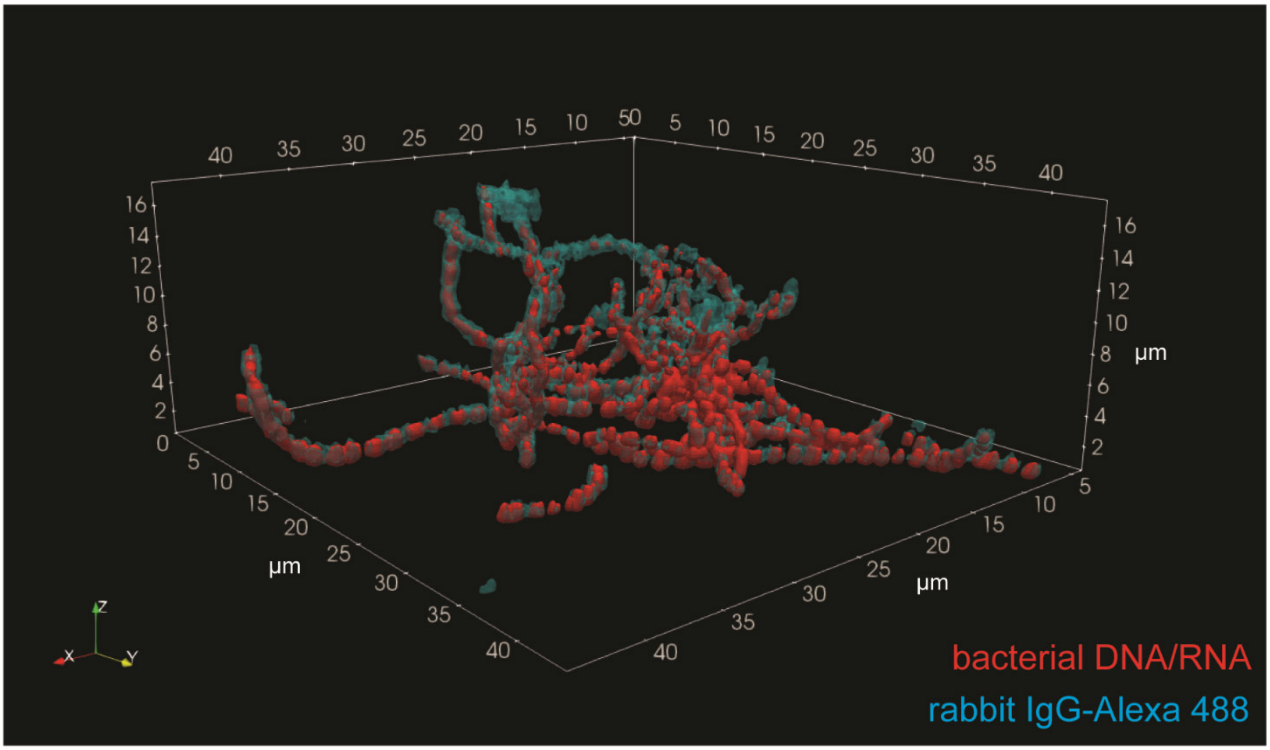

Supplementary Figure 2. Bacterial aggregation visualized by confocal laser-scanning microscopy. 3-D surface reconstruction of G361 aggregates shown in Figure 1D). Bacterial DNA/RNA is shown in red and surface bound IgG is shown in cyan.

Supplementary Figure 3

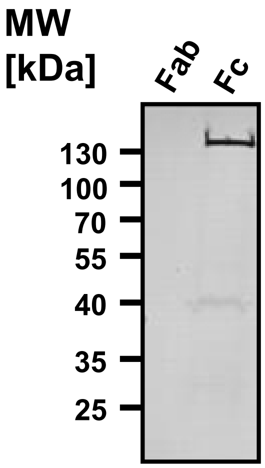

Supplementary Figure 3. IgG-Fc is sufficient for the generation of SCM-mediated protein complexes. ex formation. Recombinant SCM was co-incubated with the human IgG. fragments Fab and Fc, respectivley. Complex formation was subsequently analysed by SDS-PAGE.

Supplementary Figure 4

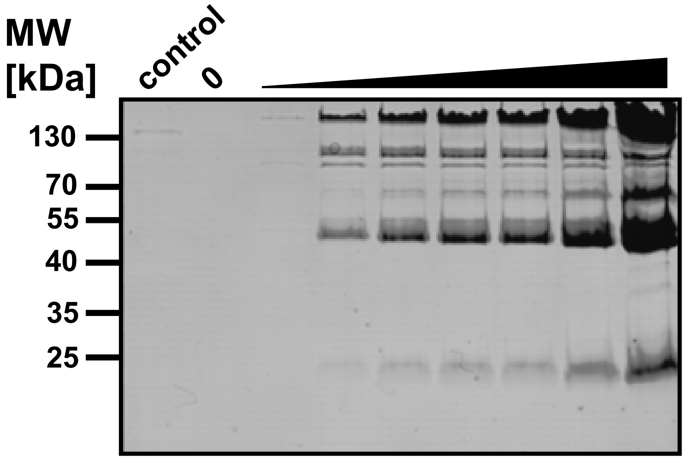

Supplementary Figure 4. Dose-dependent complex formation by SCM in human plasma. Increasing amounts of recombinant SCM protein (1 µg - 80 µg) were co-incubated with a 1:10 dilution of human plasma. The resulting protein complexes were separated by an SDS-PAGE. 20 µg of the truncated SCM fragment lacking the IgG binding region (KO173225) served as a control. 0, diluted plasma was incubated without SCM.

Supplementary Figure 5

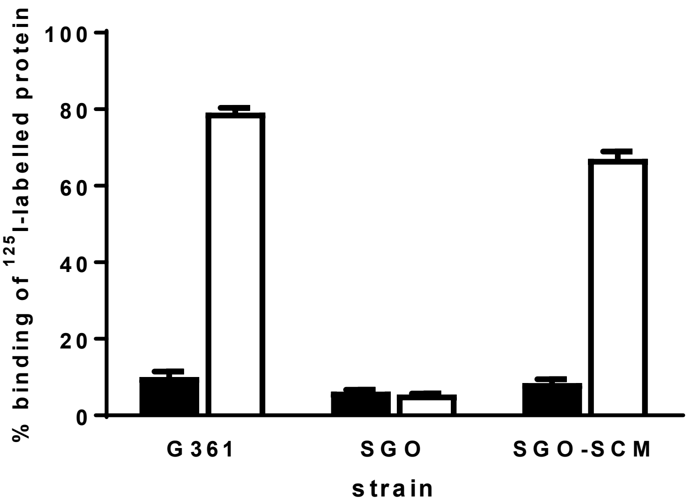

Supplementary Figure 5. SCM-mediated binding of plasminogen and fibrinogen. *S. canis* G361 and *S. gordonii* with (SGO-SCM) and without expressing SCM (SGO) were incubated with iodinated fibrinogen (black bars) and plasminogen (white bars), respectively. Results are given as mean and SD of percentage binding of totally used iodinated protein.
